# Mind the gap: the transition to hospital consultant

**DOI:** 10.1007/s40037-013-0104-x

**Published:** 2013-12-11

**Authors:** Michiel Westerman

**Affiliations:** Sassenheimstraat 78-3, 1059 BM Amsterdam, the Netherlands

**Keywords:** Transitions, Postgraduate medical education, Burnout, Generic competencies, Hospital consultant

## Abstract

Thesis defended on the 19th of December 2012 at the faculty of Medicine of the VU University Amsterdam. Promotors: Professor Fedde Scheele MD, PhD, (VUmc Amsterdam) and Professor Albert Scherpbier MD, PhD (University of Maastricht). Copromotors: Pim Teunissen, MD, PhD (University of Maastricht) and Carl Siegert MD, PhD (St. Lucas Andreas Hospital, Amsterdam).

## Introduction

Transitions can function as a lens for the merits and failings of the current medical educational system. Nevertheless, a paucity of research exists on transitions within medical education, especially concerning the transition from speciality trainee to hospital consultant. This thesis sought to clarify the processes situated within this transition. Specific research questions were: what factors in the transition to hospital consultant do doctors perceive as salient? Also, what is the influence of preparation received through speciality training on the transition to hospital consultant? And finally, what influential contextual and psychological factors can be identified within this transition?

## Methodology

I carried out a literature review and two exploratory qualitative studies, i.e., one cross-sectional and one longitudinal. Furthermore, I conducted population-based survey studies in the Netherlands and Denmark. Throughout the thesis theoretical insights from medical education literature are used as well as alternative theoretical perspectives on transitions stemming from research within social psychology, organizational studies, and pedagogy.

## Results

Newly appointed consultants perceive themselves adequately prepared for the medical and clinical aspects of their work, in particular mastery of clinical knowledge and skills. However, they report being unprepared with regard to generic competencies such as supervision, leadership, management, and handling financial issues.

This lack of preparedness within the generic competencies correlated with burnout among new hospital consultants (r = 0.15, *p* < 0.001) whereas such lack within medical competencies did not. Ten percent of the new consultants met the criteria for burnout and 18 % scored high on emotional exhaustion. Having received an increasing amount of independency during training was found to be essential for a smooth transition. Finally, the results illustrate how the transition is characterized by an intricate interplay between preparation received through training, psychological characteristics such as coping strategies and feedback seeking behaviour, and contextual factors.

## Discussion

This thesis illustrates the importance of generic competencies for adequate functioning as a hospital consultant. Furthermore, it illuminates that stressful transitions do not solely result from inadequate preparation alone and therefore cannot be prevented by alterations within the content of training programmes only. The latter would ignore the identified psychological and contextual factors within transitions. As a result, I propose a new approach to the transition from speciality trainee to hospital consultant and to transitions in the medical education continuum in general. So that transitions can then be seen not as threats, in accordance with the general perspective, but as opportunities for rapid personal and professional development. This opens up a fertile area for future research and a possible reform of speciality training.

